# The landscape of chemokine and cytokine is associated with the distinct clinical status of leprosy patients and their respective household contacts

**DOI:** 10.3389/fimmu.2024.1476450

**Published:** 2024-12-18

**Authors:** Lorena Bruna Pereira de Oliveira, Pedro Henrique Ferreira Marçal, Karolina Dias Campos, Daisy Cristina Monteiro dos Santos, Marlucy Rodrigues Lima, Olindo Assis Martins-Filho, Joaquim Pedro Brito-de-Sousa, Thais Abdala-Torres, Roberta Olmo Pinheiro, Euzenir Nunes Sarno, Jessica K. Fairley, Lucia Alves de Oliveira Fraga

**Affiliations:** ^1^ Universidade Vale do Rio Doce – Univale, Governador Valadares, MG, Brazil; ^2^ Programa Multicêntrico de Bioquímica e Biologia Molecular/PMBqBM – Universidade Federal de Juiz de Fora - UFJF, Governador Valadares, MG, Brazil; ^3^ Universidade Federal de Juiz de Fora, Campus Governador Valadares, MG, Brazil; ^4^ Grupo Integrado de Pesquisas em Biomarcadores, Instituto René Rachou, FIOCRUZ-Minas, Belo Horizonte, MG, Brazil; ^5^ Laboratório de Hanseníase, Instituto Oswaldo Cruz, Fundação Oswaldo Cruz –FIOCRUZ-RJ, Rio de Janeiro, RJ, Brazil; ^6^ Division of Infectious Diseases, Department of Medicine, School of Medicine, Emory University, Atlanta, GA, United States

**Keywords:** leprosy, Mycobacterium leprae, cytokines, chemokines, household contacts

## Abstract

**Introduction:**

Leprosy, a chronic infectious disease, is closely linked to the host immune response. According to the WHO, leprosy patients (L) and household contacts (HHC) are classified into subgroups: paucibacillary (PB) and multibacillary (MB), witch reflect the degree of infection in patients and the level of exposure of their contacts. The main goal of this study was to: i) establish a comprehensive overview of soluble mediator signatures of PBMCs upon *in vitro* antigen-specific stimuli and ii) identify whether the chemokine (CH) and cytokine (CY) signatures were associated with distinct clinical manifestations in (L) and immune response profiles in (HHC).

**Methods:**

Long-term PBMC cultures were carried out and supernatants collected for 12 CH and CY analisys by Cytometric Beads Array.

**Results and discussion:**

The CH and CY analysis, using continuous variable modeling, demonstrated that PBMCs from both L and HHC exhibited high levels of TNF upon M. leprae-stimuli. While lower production of IFN-γ were observed for L, low levels of CXCL8 was found for HHC. Soluble mediator signatures, analyzed using categorical variables, revealed that while high levels of TNF were observed for L, high levels of IFN-γ appeared as a hallmark of HHC. Overall, these analyses demonstrated that CXCL8, IFN-γ, and TNF were key markers differentiating L from HHC and endemic control (EC), especially considering the categorical analysis of the soluble mediator signatures. Data further demonstrated that higher levels of IFN-γ and lower levels CXCL8 was features associated with HHC(MB), whereas high levels of TNF were observed in both L subgroups. Moreover, data from integrative networks, based on correlation amongst soluble mediators, revealed that in M. leprae-stimuli, the number of correlations was lower in HHC(MB) compared to HHC(PB), but higher in L(MB) compared to L(PB). It was noted that the number of correlations decreased in the following order: EC > L > HHC. Our findings contribute to additional immunological features associated with L and HHC, witch can be useful complementary diagnostic/prognostic tools for classification of L and HHC, providing insights to enrich the research agenda about the hypothesis that HHC should be closely monitored as they may present a subclinical infection.

## Introduction

Leprosy is a long-term infection caused by Mycobacterium leprae and constitutes a significant global public health issue, with over 200,000 new cases reported annually worldwide (1). The disease comprises a range of clinical manifestations affecting the skin, peripheral nerves, and nasal mucosa (1, 2). As per the operational classification criteria proposed by the World Health Organization (WHO), leprosy patients can be categorized into paucibacillary (PB) or multibacillary (MB) groups based on the number of skin lesions and immune response patterns (3, 4). The timely identification and precise classification of leprosy patients are imperative for implementing appropriate multidrug therapy, constituting pivotal strategies for disease control. The current diagnostic tools for leprosy rely heavily on clinical signs and histopathological evaluation, which can lack sensitivity, particularly in subclinical cases. There is a pressing need for more sensitive and specific diagnostic methods to enable early detection and reduce transmission rates (5). Improving the efficacy of leprosy diagnosis requires developing and incorporating sensitive and specific methods (6, 7). In this context, integrating platforms and exploring complementary laboratory biomarkers are deemed significant strategies that facilitate the identification of novel targets. Furthermore, creating diagnostic tools capable of detecting subclinical leprosy in household contacts is pivotal for controlling the transmission of M. leprae. The analysis of risk factors has indicated that a high bacillary load in index cases constitutes a significant variable linked to the occurrence of leprosy amongst household contacts (8). The immune response profile to the M. leprae antigens has been shown to differentiate leprosy patients and household contacts from healthy controls (3, 9). Previous studies conducted by our research team have demonstrated the relevance of combining immunological approaches and genetic biomarkers with artificial intelligence methodologies to improve the diagnosis of leprosy and identify household contacts at risk of developing the disease (10–14). These studies identified key immunological and genetic markers, such as TNF, IFN-γ, and a single nucleotide polymorphism (SNP) in the TLR4 gene, which can help monitor household contacts at risk, as cytokine levels vary according to the clinical form of leprosy. The SNP in TLR4 is an indicator of susceptibility, allowing for early diagnosis. Additionally, the studies detected M. leprae DNA in biological samples from asymptomatic contacts who developed the disease after two years of follow-up. Moreover, these approaches have proven helpful in categorizing leprosy patients and monitoring household contacts. Interestingly overall studies have proposed that distinct patterns of immune response can influence the outcome of leprosy and the development of clinical disease in household contacts, suggesting that the assessment of cell-mediated immune response by examining chemokines and cytokines profiles within T-cell subsets is a relevant approach for follow-up purposes (15). The present study proposes a complementary approach to delineate the comprehensive landscape of chemokine and cytokine signatures and establish correlations with distinct clinical manifestations among leprosy patients and household contacts. Twelve cytokines and chemokines were measured in PBMC supernatants, providing a comprehensive view of the immune response profiles across the different study groups. Our hypothesis suggests that the signatures and network patterns of chemokines and cytokines are linked to distinct clinical classifications of leprosy and household contacts. The comprehensive profiles of chemokines and cytokines observed in this study provide further insights into the immunological events, identifying biomarkers such as CXCL8, TNF, and IFN-γ, which may help differentiate between various exposure levels (accoding to the index case operational classification) in household contacts. These findings enhance our understanding of how prolonged exposure to M. leprae in endemic populations, particularly among household contacts, influences immune responses. This knowledge can be applied to future clinical studies and improve patient management strategies.

## Methods

### Ethics statement

This study was submitted and approved by the Ethics Committee from Universidade Federal de Juiz de Fora—UFJF (Research Protocol CAAE #56863016.6.1001.5147). All participants or their next of kin read and signed a free and informed consent form before inclusion in the study. Parents/guardians provided consent on behalf of minor participants.

### Study design and participants

The present study is an exploratory observational investigation carried out in Governador Valadares Municipality, Minas Gerais, Brazil, considered a hyperendemic area for leprosy (1.9 cases/10,000 inhabitants), based on the overall prevalence observed in Minas Gerais (0.5 cases/10,000 inhabitants) and Brazil (1.2 cases/10,000 inhabitants) (1). The participants were contacted at the Reference Center for Endemic Diseases and Special Programs (CREDEN-PES) outpatient unit, Department of Public Health, Governador Valadares, MG, Brazil.

**Figure 1:**
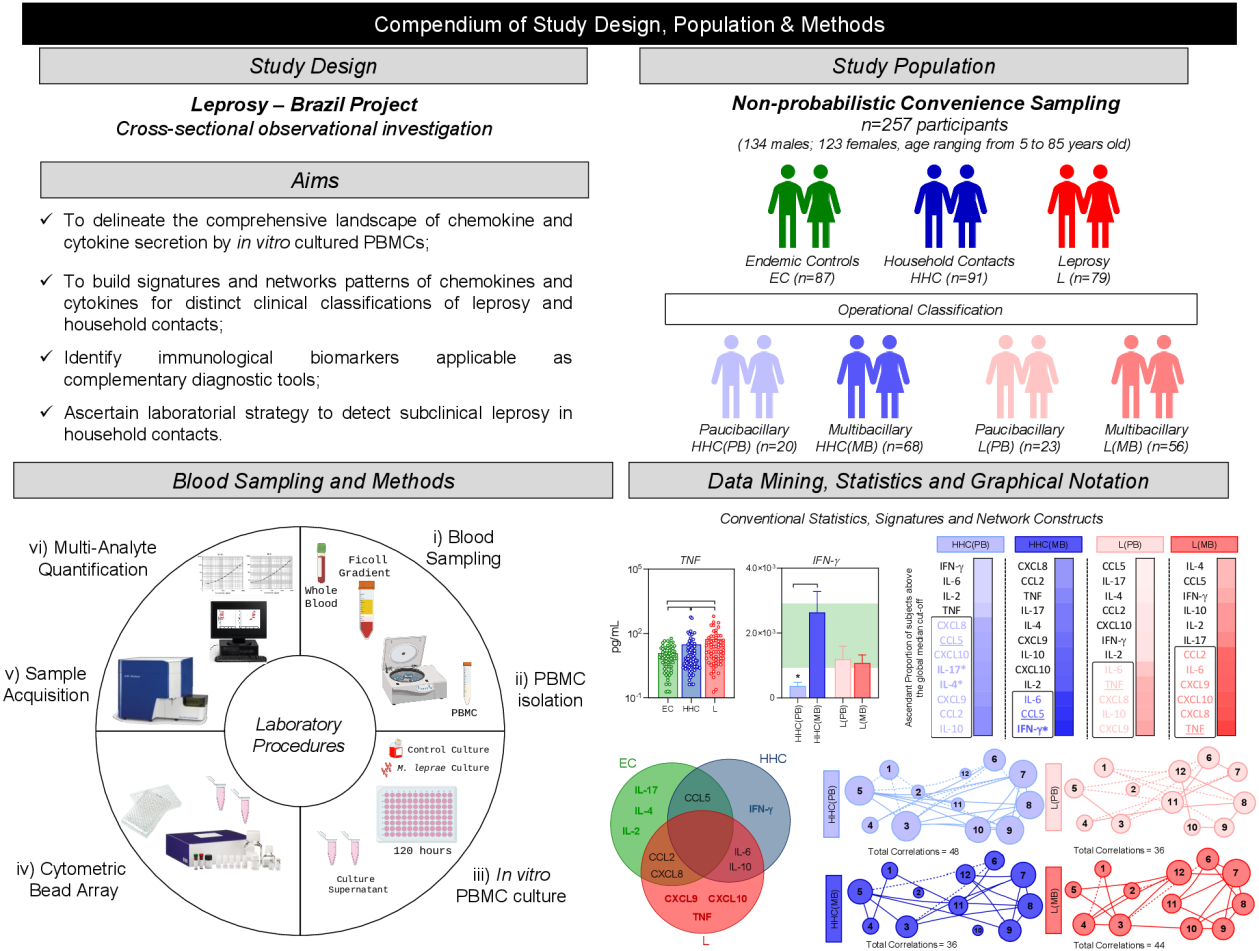
Compendium of study design, population & methods. This is an exploratory observational investigation enrolling a total of 257 participants, selected as a non-probabilistic convenience sampling to characterize the profile of soluble immune mediators produced by PBMC upon *in vitro* culture. The study population comprises three major groups of subjects, referred as: endemic healthy controls (EC – 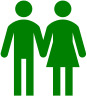, n= 87), household contacts of leprosy patients (HHC – 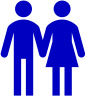, n=91) and Leprosy patients (L, – 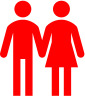, n=79). HHC and L subjects were further classified into subgroups named paucibacillary and multibacillary, according to operational classification records and referred as to: [HHC(PB) – 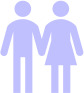, n=20], [HHC(MB) – 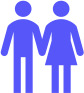, n=78], [L(PB) – 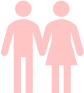, n=23] and [L(MB) – 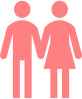, n=56]. Heparinized whole blood samples (10mL) from were obtained from each participant and used to isolate PBMCs for experimental procedures to quantify soluble mediators by Cytometric Bead Array to quantify chemokines (CXCL8, CCL2, CXCL9, CCL5, CXCL10) and cytokines (IL-6, TNF, IFN-γ, IL-17, IL-4, IL-10 and IL-2) in cell culture supernatants obtained in the absence on exogenous stimuli and in the presence of M. leprae stimuli. Distinct approaches were employed for data mining and statistics, including: conventional statistics, soluble mediator signatures and networks. Systems immunology tools were employed to assemble integrative network, color maps and fold change constructs.

This study comprises a cross-sectional investigation, named Leprosy – Brazil Project, that enrolled a total of 257 participants of both genders (52% males and 48% females), ages ranging from 5 to 85 years old, selected as a non-probabilistic convenience sampling, encompassing: 91 household contacts of leprosy patients (HHC, 38% males and 62% females, median age = 33 years old, ranging from 5 to 85 years old); 79 patients (L, 56% males and 44% females, median age = 47 years old, ranging from 6 to 80 years old) and 87 endemic healthy controls (EC, 52% males and 48% females, median age = 46 years old, ranging from 10 to 83 years old). According to the Brazilian Guidelines (16), the operational classification of leprosy cases is based on the number of skin lesions, considering the following criteria: Paucibacillary (PB) – cases presenting up to five lesions; Multibacillary (MB) – cases with more than five skin lesions. Although, positive baciloscopic index (BI≠0) classifies the cases as MB, negative baciloscopic index (BI=0) does not necessarily classify the patient as PB, as it depends on the number of lesions the case presents. Moreover, the number of lesions lower than five does not necessarily classify the case as PB, since cases with lesion number up to five, but presenting baciloscopic index (BI≠0) is classified as MB. Operational classification records were available for 88 out of 91 (96%) HHC. The bacilloscopic index values (BI=0) for index cases of HHC(PB) and number of lesions (>5) for index cases of HHC(MB) were obtained from medical records. Based on the operational classification records, the HHC subjects (n=88 out of 91) and all L patients (n=79) were further classified into subgroups, which reflect the degree of infection in patients and the level of exposure of their contacts. In our study these subgroups are referred as to: HHC(PB), (n=20) – household contacts of paucibacillary leprosy patients; HHC(MB), (n=78) – household contacts of multibacillary leprosy patients; L(PB), (n=23) – paucibacillary leprosy patients; L(MB), (n=56) – multibacillary leprosy patients. All patients were untreated and did not present reactions at the time of sample collection, ensuring that the immune response observed was not influenced by ongoing leprosy treatment. For operational purpose, household contacts (HHC) are defined as an individual who resides or has resided, or who lives or has lived with a leprosy patient within the household, during the five years prior to the diagnosis of the disease, whether a family member or not (16). **Table 1** provides the study population’s major demographic features and clinical laboratory records. **Figure 1** summarizes the compendium of study design, population & methods.

**Table 1:** Demographic features and clinical-laboratorial records of the study population.

Parameters	EC (n=87)	HHC* (n=91)	L (n=79)	HHC(PB)§ (n=20)	HHC(MB)§ (n=68)	L(PB) (n=23)	L(MB) (n=56)
Age^#^ median (min-max)	46 (10-83)	33 (5-85)	47 (6-80)	37 (7-79)	30 (5-85)	39 (10-60)	49 (6-80)
Sex (%)							
Males	52	38	56	40	38	48	59
Females	48	62	44	60	62	52	41
Number of Lesion median (min-max)	–	–	2 (1-10)	NA	>5	1 (1-4)	3 (1-10)
Bacilloscopic Index mean (min-max)	–	–	0.8 (0-4.5)	0 (0-0)	NA	0 (0-0)	1.1 (0-4.5)
WBC (cells/mm^3^)	7,200	7,400	6,800	6,950	7,600	7,900	6,500
Neu (%)	63	61	58	58	61	58	58
Mon (%)	4	5	5	5	4	5	5
Lym (%)	28	31	31	33	30	31	32
RBC (x10^6^/mm^3^)	4.7	4.6	4.4	4.7	4.6	4.5	4.4
Hemoglobin (g/dL)	13.8	13.3	13.3	13.6	13.3	13.2	13.3
Hematocrit (%)	43	41	41	41	41	41	41
Platelet (count x10^3^/mm^3^)	244	247	234	220	252	249	220

EC, Endemic healthy controls; HHC, Household contacts; L, Leprosy patients. * Operational classification records were available for 88 HHC volunteers. HHC(PB), household contacts of paucibacillary leprosy patients. HHC(MB), household contacts of multibacillary leprosy patients; L(PB), paucibacillary leprosy patients; L(MB), multibacillary leprosy patients. § Bacilloscopic Index values for index cases of HHC(PB) and Number of Lesions for index cases of HHC(MB) were obtained from medical records. NA, Not Available medical records. ^#^ Age is expressed in years old. WBC, White blood cells; Neu, neutrophils; Mon, Monocytes; Lym, lymphocytes; RBC, Red blood cells. Laboratorial records are expressed as median values.

### Peripheral blood sampling and PBMC culture *in vitro*

Heparinized whole blood samples (10mL) were obtained from each participant and used to isolate peripheral blood mononuclear cells (PBMC) for *in vitro* cultures and further quantification of chemokine and cytokine in culture supernatant. PBMC were separated by Ficoll Hypaque cushion gradient, washed twice, and resuspended in RPMI-1640, supplemented with 10% fetal bovine serum, 2mM L-glutamine, penicillin (100U/mL) and streptomycin (100mg/mL). Cell aliquots (2x10^5^/well) were cultured in duplicates on flat-bottom 96-well plates at 37°C, 5% of CO_2_ in a humidified incubator. Parallel culture batches were carried out without exogenous stimuli (unstimulated culture) and in the presence of 10 irradiation-killed M. leprae bacilli/cell (M. leprae-stimulated culture). The M. leprae bacilli were prepared at Instituto Lauro de Souza Lima (São Paulo, Brazil), as previously described (17). Culture supernatants were harvested after five days, centrifugated to remove cells/debris, aliquoted, and stored at -80°C until processing for soluble mediator analysis. We selected day 5 for supernatant collection based on prior studies showing this time point best captures peak cytokine and chemokine production in PBMC cultures stimulated with antigen, as it is the most representative for detecting immune responses to M. leprae (14).

### Soluble mediator quantification by bead-based immunoassays

Quantitative analysis of chemokines and cytokines in cell culture supernatants was performed by Cytometric Bead Array (Human Chemokine Kit for CXCL8, CCL2, CXCL9, CCL5 and CXCL10 and Human Cytokine Flex Set kit for IL-6, TNF, IFN-γ, IL-17, IL-4, IL-10 and IL-2) purchased from BD Bioscience Pharmingen (San Diego, CA, USA) according to the manufacturer instructions. 400 events/chemokine or cytokine-specific beads were acquired on a FACSVerse™ Bioanalyzer (BD Bioscience, San Jose, CA, USA). Data was analyzed using the FCAP Array software (Soft Flow, Inc., St. Louis Park, MN, United States). The results were expressed in pg/mL based on the standard curves using the 5-parameter logistic regression model.

### Statistical analysis

Statistical analysis was carried out using the Prism GraphPad software (version 10.2.0, San Diego, California, USA). Considering the non-parametric distribution of data sets, comparative analysis amongst groups was carried out by the Kruskal-Wallis test followed by Dunn’s post-test for multiple comparisons amongst EC vs. HHC vs. L as well as amongst EC vs. HHC(PB) vs. HHC(MB) vs. L(PB) vs. L(PB) subgroups. In all cases, significant differences were considered at p<0.05.

Additional descriptive analysis of soluble mediators, referred to as signatures, was carried out as previously reported by Cunha et al. (14) and modified as follows: the intrinsic median values for each cytokine and chemokine were derived based on their distribution under specific culture conditions (unstimulated culture or M. leprae-stimulated culture). Thus, two distinct medians were obtained for each biomarker, depending on the stimulus, providing a robust baseline for classifying the subjects into low and high expression groups. Continuous variables expressed in pg/mL were converted into categorical data using the intrinsic median values of unstimulated culture (CXCL8 = 6,194; CCL2 = 3,669; CXCL9 = 778; CCL5 = 707; CXCL10 = 1,351; IL-6 = 10,135; TNF = 8.1; IFN-γ = 560; IL-17 = 7.2; IL-4 = 0.8; IL-10 = 42 and IL-2 = 13 pg/mL) or M. leprae stimulated culture (CXCL8 = 5,460; CCL2 = 3,518; CXCL9 = 852; CCL5 = 641; CXCL10 = 1,248; IL-6 = 10,057; TNF = 8.2; IFN-γ = 456; IL-17 = 6.3; IL-4 = 1.0; IL-10 = 45 and IL-2 = 16 pg/mL) as the cut-off to identify volunteers with low and high levels of soluble mediators. Following, the proportion of subjects (%) with chemokine and cytokine levels above the intrinsic cut-off was calculated for each study group or subgroup. The chemokines and cytokines with the proportion of subjects above the 50^th^ percentile was underscored and considered as increased levels. The chemokines and cytokines signatures were assembled as ascendant signatures, and Venn diagrams were constructed to identify common and selective soluble mediators amongst groups.

Integrative networks of chemokines and cytokines were built based on correlation analysis (Spearman rank tests) between pairs of soluble mediators. Only significant correlations (p<0.05) were employed to construct networks (chemokines and cytokines) using the open-source Cytoscape software (available at https://cytoscape.org). The networks were assembled using cluster layouts with nodes representing each chemokine and cytokine and connecting lines identifying positive (“r” scores >0, continuous line) or negative (“r” scores <0, dashed line) correlations. The node sizes are proportional to the correlations between pairs of soluble mediators. Comparative analysis amongst groups was carried out considering the total number of correlations. Colormap analysis of integrative networks was used to illustrate the comparison amongst groups using a color key based on the percentile distribution (10^th^/50^th^/90^th^) of correlation numbers calculated for soluble mediator, chemokine, and cytokine clusters or the total number of correlations.

An additional analysis was performed to summarize the significant changes observed in soluble mediators amongst groups. For this purpose, the fold changes in chemokines and cytokines concentrations in PBMC culture supernatants from HHC and L were calculated using the relative ratio to EC (Fold Change in concentration = HHC/EC or L/EC). To accomplish the comparative analysis of HHC and L subgroups according to operational classification, fold changes were calculated about PB subjects [Fold Change in concentration HHC(MB)/HHC(PB) or L(MB)/L(PB)]. Log10 of Fold Change values (magnitude) were combined with the -Log10 p values (significance) obtained by Dunn’s post-test for multiple comparisons.

Differences in chemokines and cytokines signatures were assessed for EC and PB subjects [Delta in % = %HHC – %EC or %L – % EC; %HHC(MB) – %HHC(PB) or %L(MB) – %L(PB)]. The Log10 of Delta values (magnitude) was combined with the -Log10 p values (significance) obtained by Chi-square comparisons.

Differences in chemokines and cytokines network correlations were assessed according to EC and PB subjects [Delta in correlation numbers = HHC – EC or L – EC; HHC(MB) – HHC(PB) or L(MB) – L(PB)]. Delta values (magnitude) were combined with the % of correlations from EC or PB (threshold = decrease or increase > 33%).

## Results

### Panoramic overview of chemokines and cytokines secretion by *in vitro* cultured PBMCs from endemic controls, household contacts, and leprosy patients

**Figure 2:**
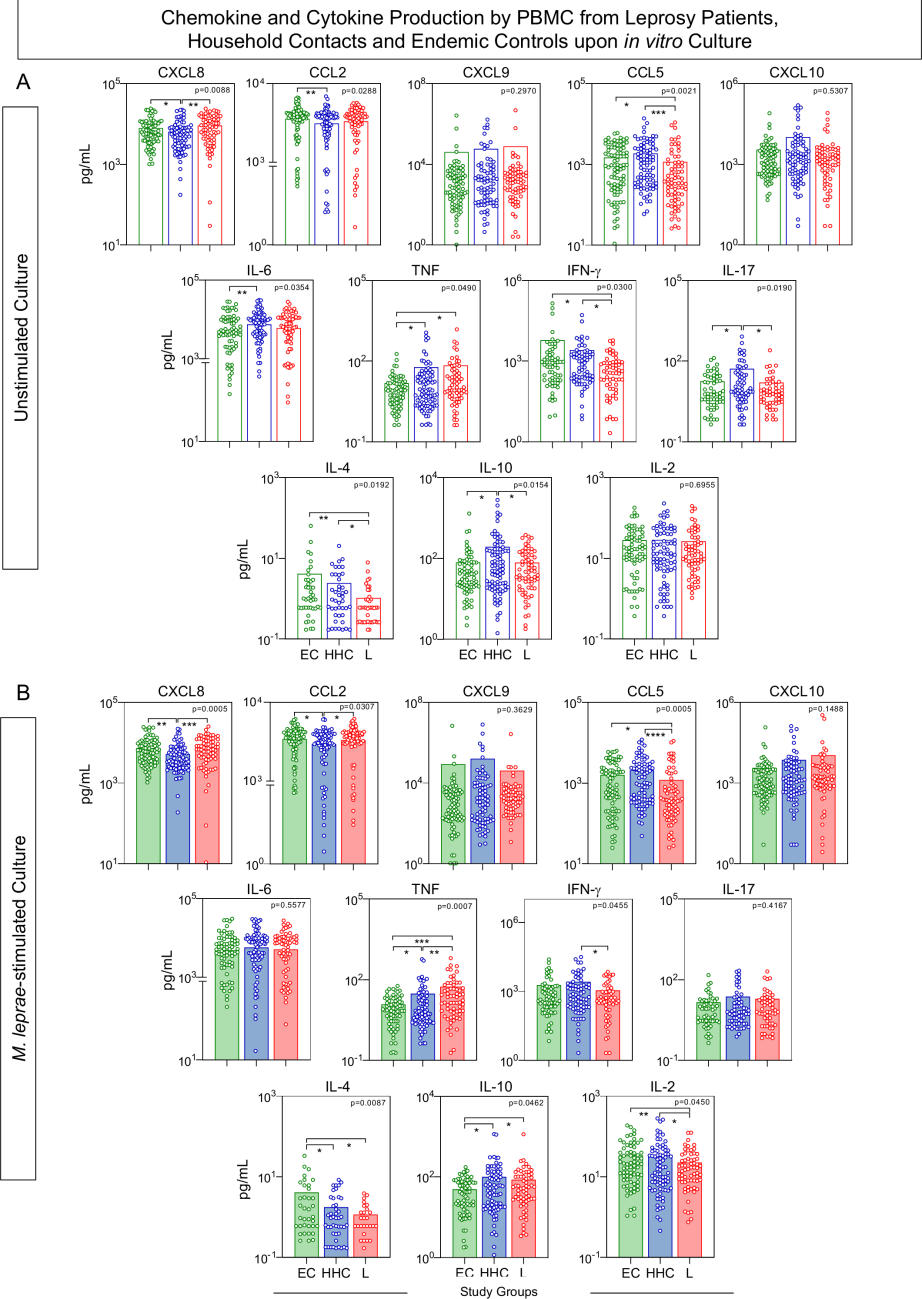
Chemokine and cytokine production by PBMC from Leprosy Patients, Household Contacts, and Endemic Controls upon *in vitro* culture. The levels of chemokines (CXCL8, CCL2, CXCL9, CCL5, CXCL10) and cytokines (IL-6, TNF, IFN-γ, IL-17, IL-4, IL-10 and IL-2) were measured in the supernatant from *in vitro* cultured PBMC from leprosy patients (L = 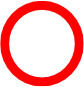, n=79), household contacts (HHC = 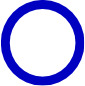, n=91) and endemic controls (EC = 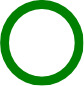, n=87). Data were obtained **(A)** in the absence of exogenous stimuli (Unstimulated Culture = open bars) and **(B)** in the presence of M. leprae antigen stimuli (M. leprae-stimulated Culture = filled bars). Cytometric Beads Array (CBA) performed quantitative analysis of chemokines and cytokines according to manufacturer instructions. The results are expressed in pg/mL and presented as a scatter distribution of individual values over bars showing mean values. Comparative analysis amongst groups was carried out by Kruskal-Wallis test followed by Dunn’s post-test for multiple comparisons amongst EC vs HHC vs L subgroups. In all cases, significant differences were considered at p<0.05. Kruskal-Wallis p values are provided for each parameter and the significant differences amongst groups identified by Dunn’s post-test are indicated by connecting lines and * (p<0.05), ** (p<0.01), *** (p<0.001) and **** (p<0.0001).

In order to characterize the soluble mediator profile of PBMC from EC, HHC, and L, the levels of chemokines (CXCL8, CCL2, CXCL9, CCL5, CXCL10) and cytokines (IL-6, TNF, IFN-γ, IL-17, IL-4, IL-10, and IL-2) were measured in the supernatant collected upon *in vitro* culture. **Figure 2** illustrates the analysis of soluble mediators, expressed as the concentration of each biomarker in pg/mL, treated as continuous variables. Data from cultures without exogenous stimuli (unstimulated cultures) revealed that household contacts (HHC) exhibited lower levels of CXCL8 and CCL2 but higher production of IL-6, TNF, IL-17, and IL-10 when compared to endemic controls (EC). Additionally, the leprosy patients (L) group showed elevated TNF levels, but reduced secretion of CCL5, IFN-γ, and IL-4, compared to EC (**Figure 2A**).

In M. leprae-stimulated cultures, HHC maintained lower production of CXCL8 and CCL2 while displaying higher levels of TNF and IL-10, with a reduction in IL-4 production, compared to EC. Conversely, the L group showed consistently higher TNF levels and decreased production of IFN-γ and IL-4, along with lower levels of CCL5 and IL-2, and elevated IL-10 levels, when compared to EC (**Figure 2B**).

### Signature of chemokine and cytokine triggered by *in vitro* cultured PBMC from leprosy patients, household contacts, and endemic controls

**Figure 3:**
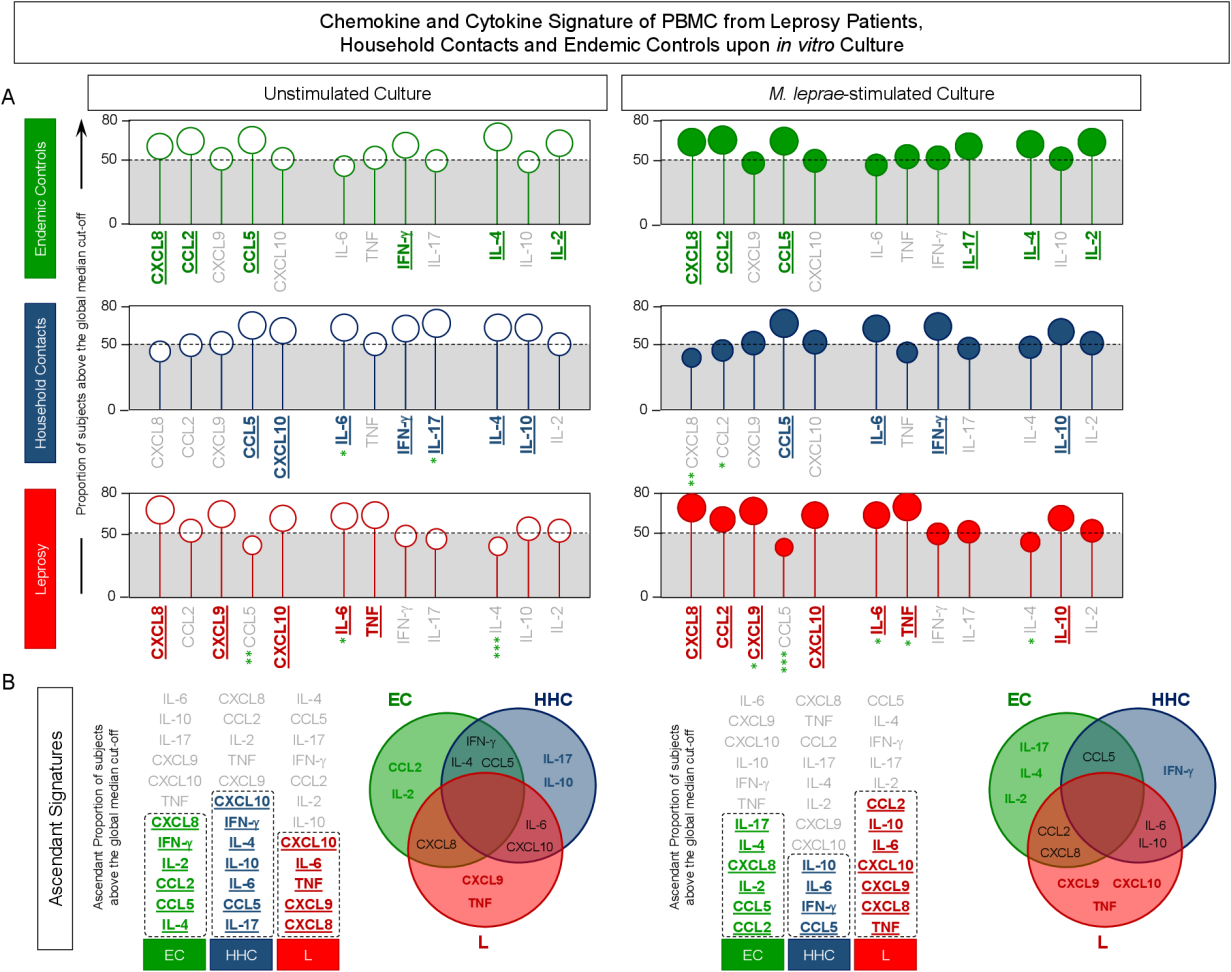
Signature of chemokine and cytokine produced by PBMC from Leprosy Patients, Household Contacts and Endemic Controls upon *in vitro* culture. The levels of chemokines (CXCL8, CCL2, CXCL9, CCL5, CXCL10) and cytokines (IL-6, TNF, IFN-γ, IL-17, IL-4, IL-10, and IL-2) were measured in the supernatant from *in vitro* cultured PBMC from leprosy patients (L = 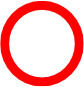, 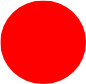, n=79), household contacts (HHC = 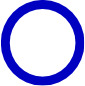, 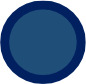, n=91) and endemic controls (EC = 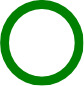, 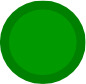, n=87). Data were obtained in the absence of exogenous stimuli (Unstimulated Culture = open circles) and the presence of M. leprae antigen stimuli (M. leprae-stimulated Culture = filled circles). Cytometric Beads Array (CBA) performed quantitative analysis of chemokines and cytokines according to manufacturer instructions. The results are reported as chemokine and cytokine signatures as described in the methods. Continuous variables expressed in pg/mL were converted into categorical data using the intrinsic median values of unstimulated culture or M. leprae stimulated culture as the cut-off to identify volunteers with low and high soluble mediators as described in methods. **(A)** The proportion of subjects (%) with chemokine and cytokine levels above the intrinsic cut-off was calculated for each study group, and data are presented as lollipop charts. The chemokines and cytokines with the proportion of subjects above the 50^th^ percentile were underscored and considered as increased levels were underscored by color font format. Comparative analysis between HHC vs EC and L vs EC was carried out by Chi-square and significant differences indicated by * (p<0.05), ** (p<0.01) and *** (p<0.001). **(B)** The chemokines and cytokines signatures were further assembled as ascendant signatures, and Venn diagrams were constructed to identify common and selective soluble mediators amongst groups.

The soluble mediators measured in the supernatant from *in vitro* cultured PBMC were further assessed as chemokines and cytokines signatures. For this purpose, the levels of soluble mediators expressed in pg/mL were converted into categorical data as described in Methods, and the results are shown in **Figure 3**. Data analysis of the proportion of subjects above the 50^th^ cut-off allowed the identification of clusters of soluble mediators featuring each study group (**Figure 3A**). A Venn Diagram analysis identified common and selective soluble mediators amongst EC, HHC, and L groups (**Figure 3B**). The results from Unstimulated cultures demonstrated that the production of CCL2 and IL-2 was the hallmark of EC. The hallmark of HHC was the increased production of IL-17 and IL-10, while TNF production represents discriminant attributes observed in L. The signature analysis of categorical data further identified CXCL9 as a selective attribute of the L group (**Figure 3B**, Left panel).

The Venn diagram analysis of M. leprae-stimulated cultures reveals IL-17, IL-4, and IL-2 as selective attributes for EC, IFN-γ for HHC and CXCL9, CXCL10, and TNF for the L group (**Figure 3B**).

These findings underscore that upon M. leprae-stimuli, IFN-γ-upregulation is observed in household contacts, and CXCL10 is observed in leprosy patients.

### Networks of chemokine and cytokine elicited *in vitro* by cultured PBMCs from endemic controls, household contacts, and leprosy patients

**Figure 4:**
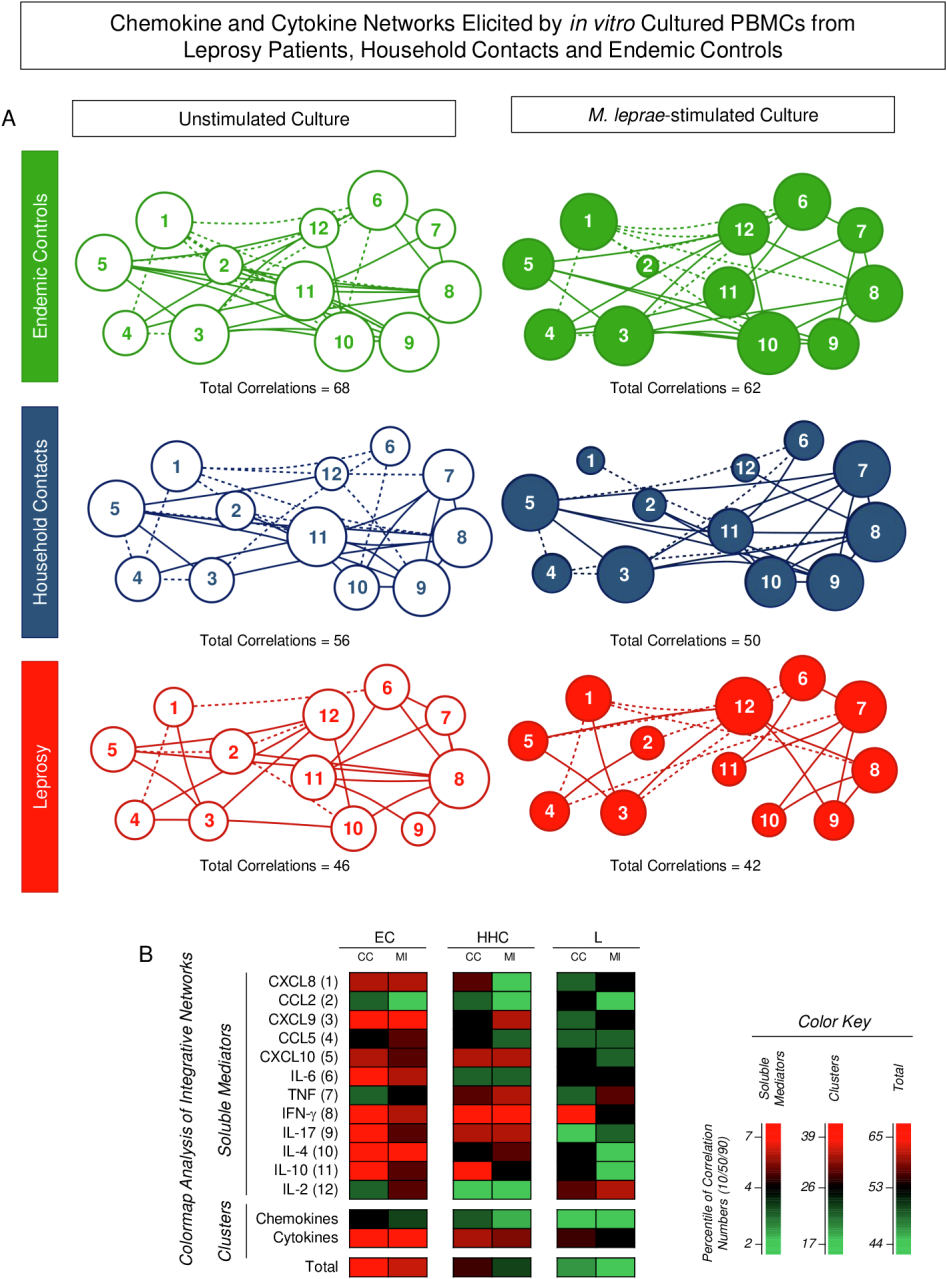
Integrative networks of chemokine and cytokine elicited by *in vitro* cultured PBMCs from Leprosy Patients, Household Contacts, and Endemic Controls. The levels of chemokines (CXCL8, CCL2, CXCL9, CCL5, CXCL10) and cytokines (IL-6, TNF, IFN-γ, IL-17, IL-4, IL-10, and IL-2) were measured in the supernatant from *in vitro* cultured PBMC from leprosy patients (L =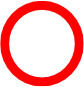; 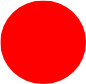, n=79), household contacts (HHC = 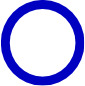; 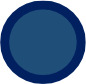, n=91) and endemic controls (EC = 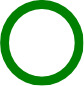, 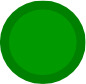, n=87). Data were obtained in the absence of exogenous stimuli (Unstimulated Culture = open circles) and the presence of M. leprae antigen stimuli (M. leprae-stimulated Culture = filled circles). Cytometric Beads Array (CBA) performed quantitative analysis of chemokines and cytokines according to manufacturer instructions. Integrative networks were built based on correlation analysis (Spearman rank tests) between pairs of soluble mediators, and significant correlations (p<0.05) were employed to construct networks using the open-source Cytoscape software as described in the methods. **(A)** The networks were assembled using cluster layouts with nodes representing each chemokine and cytokine (numbered as provided in the figure) and connecting lines identifying positive (“r” scores >0, continuous line) or negative (“r” scores <0, dashed line) correlations. The node sizes are proportional to the correlations between pairs of soluble mediators. Comparative analysis amongst groups was carried out considering the total number of correlations. **(B)** Colormaps analysis of integrative networks illustrates the comparison amongst groups using a color key based on the percentile distribution (10^th^/50^th^/90^th^) of correlation numbers calculated for soluble mediator, chemokine, and cytokine clusters or the total number of correlations.

Integrative networks were assembled based on the correlation profile between pairs of soluble mediators to investigate further the interplay amongst chemokines and cytokines in the PBMC culture microenvironment (**Figure 4**). Data analysis showed that, regardless of the culture condition, the number of correlations displayed a decrease in HHC and L as compared to EC. Moreover, the M. leprae-stimuli led to a decrease in the number of correlations between soluble mediators as compared to the Unstimulated culture (EC = 68 → 62; HHC = 56 → 50 and L = 46 → 42) (**Figure 4A**). Color map analysis further illustrated that downregulation was universally observed in the total number of correlations, intra-cluster connectivity, and the analysis of single patterns of most soluble mediators (**Figure 4B**). Detailed description of correlations between pairs of chemokines and cytokines together with the Spearman rank “r” scores are provided in the **Supplementary Figure 1**.

### Summary of changes in chemokine and cytokine profile produced by *in vitro* cultured PBMCs from endemic controls, household contacts, and leprosy patients

**Figure 5:**
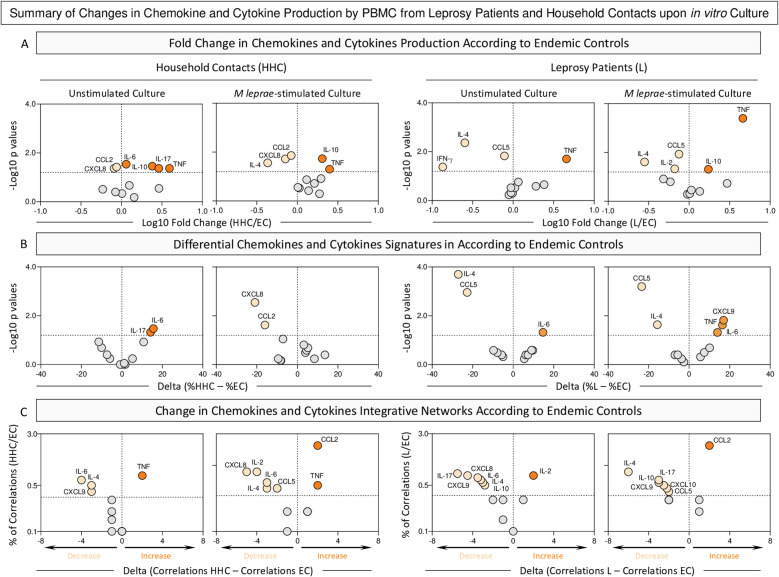
Summary of changes in chemokine and cytokine production by PBMC from Leprosy Patients and Household Contacts upon *in vitro* culture. The levels of chemokines (CXCL8, CCL2, CXCL9, CCL5, CXCL10) and cytokines (IL-6, TNF, IFN-γ, IL-17, IL-4, IL-10 and IL-2) were measured in the supernatant from *in vitro* cultured PBMC from leprosy patients (L, n=79), household contacts (HHC, n=91) and endemic controls (EC, n=87). Data were obtained in the absence of exogenous stimuli (Unstimulated Culture) and the presence of M. leprae antigen stimuli (M. leprae-stimulated Culture). Cytometric Beads Array **(CBA)** performed quantitative analysis of chemokines and cytokines according to manufacturer instructions. **(A)** Major changes observed in soluble mediators amongst groups were summarized considering the fold changes in concentrations detected in PBMC culture supernatants from HHC and L according to EC (Fold Change in concentration = HHC/EC or L/EC). Log10 of Fold Change values (magnitude) was combined with the -Log10 p values (significance) obtained by Dunn’s post-test for multiple comparisons. **(B)** Differences in chemokines and cytokines signatures were assessed according to EC (Delta in % = %HHC – %EC). Log10 of Delta values (magnitude) was combined with the -Log10 p values (significance) obtained by Chi-square comparisons. **(C)** Differences in chemokines and cytokines network correlations were assessed according to EC (Delta in correlation numbers = HHC – EC or L – EC). Delta values (magnitude) were combined with the % of correlations from EC (threshold = decrease or increase > 33%). In all cases, significant decrease (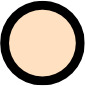), increase (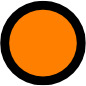), or non-significant changes (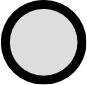) are highlighted on each chart distribution.

**Figure 5** provides a snapshot of the significant changes observed in the chemokine and cytokine profile elicited by the *in vitro* culture of PBMCs. Distinct approaches were employed to scrutinize details of soluble mediator settings in HHC and L groups. For this purpose, the fold change in concentration magnitude, along with shifts in the soluble mediator signatures and integrative networks, were assessed according to EC.

The analysis of fold changes in soluble mediator concentrations demonstrated that regardless of the culture condition, decreased levels of CXCL8 and CCL2 and increased levels of TNF and IL-10 were observed for HHC. The M. leprae-stimuli downregulated the levels of IL-6 and IL-17. In the L group, consistent decrease of CCL5 and IL-4 was observed despite the culture condition. At the same time, IL-10 is observed in L patients only upon M. leprae stimuli (**Figure 5**, top panels).

Data from the soluble mediator signatures further corroborates the loss of IL-6 and IL-17 in HHC upon antigen stimulation. The analysis of L group brings about the insight that decreased levels of CCL5 and IL-4 are consistently observed in L regardless of the culture condition (**Figure 5**, middle panels).

The scrutiny of integrative networks supports the overall downregulation in the correlation numbers between soluble mediators in HHC and L as compared to EC (**Figure 5**, bottom panels).

An integrative analysis of changes in chemokines and cytokines highlights distinct immune response profiles that differentiate HHC and L from EC (**Supplementary Figure 2**). Key biomarkers emerge, defining the common and selective immunological landscapes of each group. An increase in TNF stands out as common marker of inflammation in HHC group across all culture conditions. Conversely, a decrease of CCL5 and IL-4 appears prominently in the L group, regardless the culture condition (**Supplementary Figure 2**).

### Analysis of chemokines and cytokines secretion by *in vitro* cultured PBMCs from household contacts and leprosy patients categorized according to operational classification

**Figure 6:**
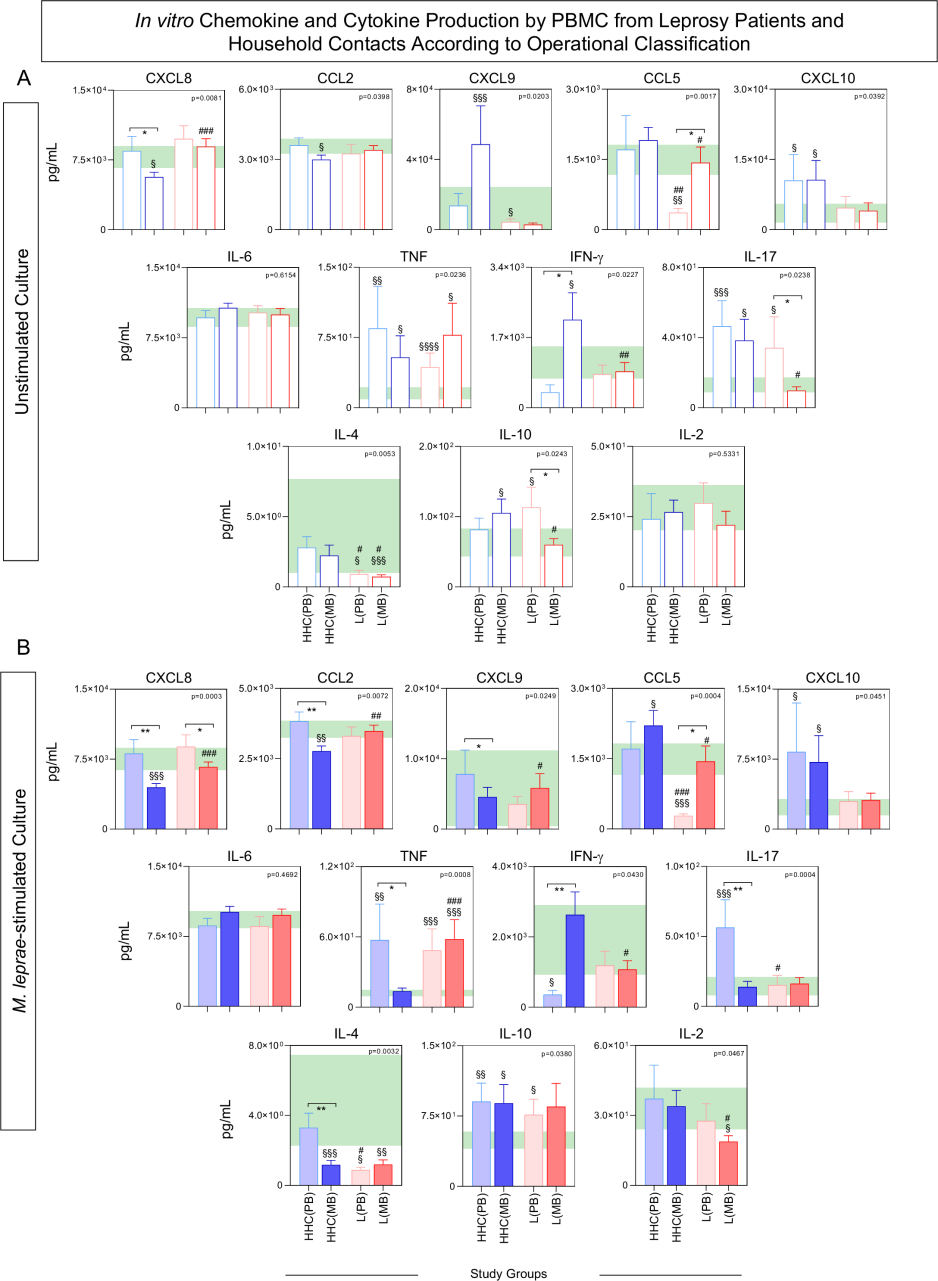
*In vitro* chemokine and cytokine production by PBMC from Leprosy Patients and Household Contacts according to operational classification. The levels of chemokines (CXCL8, CCL2, CXCL9, CCL5, CXCL10) and cytokines (IL-6, TNF, IFN-γ, IL-17, IL-4, IL-10 and IL-2) were measured in the supernatant from *in vitro* cultured PBMC from leprosy patients [L(PB) = 
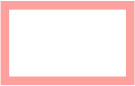
; 
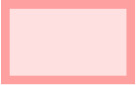
, n=23 and L(MB) = 
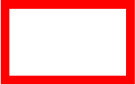
; 
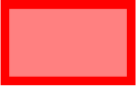
, n= 56] and household contacts [HHC(PB) = 
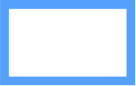
; 
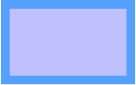
, n=20 and HHC(MB) = 
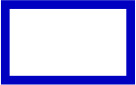
; 
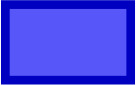
, n= 68] subgroups for comparisons with endemic controls (EC = green reference 95%CI range, n=87). Data were obtained **(A)** in the absence of exogenous stimuli (Unstimulated Culture = open bars) and **(B)** in the presence of M. leprae antigen stimuli (M. leprae-stimulated Culture = filled bars). Cytometric Beads Array (CBA) performed quantitative analysis of chemokines and cytokines according to manufacturer instructions. The results are expressed in pg/mL and presented in bar charts showing mean values and standard error. Comparative analysis amongst groups was carried out by Kruskal-Wallis test followed by Dunn’s post-test for multiple comparisons amongst EC vs HHC(PB) vs HHC(MB) vs L(PB) vs L(PB) subgroups. In all cases, significant differences were considered at p<0.05. Kruskal-Wallis p values are provided for each parameter and the significant differences amongst groups identified by Dunn’s post-test are indicated by § for comparisons with EC, # for intergroup comparisons and connecting lines with * for MB vs PB intragroup comparisons. In all case, the number of symbols (1, 2, 3 and 4) indicated the power of p values (p<0.05, p<0.01, p<0.001 and p<0.0001, respectively).

To further characterize the chemokine and cytokine profiles of PBMCs from distinct subgroups of HHC and leprosy patients (L), subjects were stratified based on operational classification into the following groups: HHC(PB), HHC(MB), L(PB), and L(MB). The results are presented in **Figure 6**.

In unstimulated cultures, comparative analysis revealed that HHC(MB) exhibited higher levels of IFN-γ and lower levels of CXCL8 compared to HHC(PB). Conversely, L(PB) showed elevated levels of IL-17 and IL-10, alongside reduced levels of CCL5, when compared to L(MB) (**Figure 6A**).

In M. leprae-stimulated cultures, HHC(PB) displayed higher levels of CXCL9, TNF, and IL-17, along with lower IFN-γ levels, while L(MB) demonstrated lower levels of CXCL8, CCL2, and IL-4 (**Figure 6B**).

### Functional signatures elicited by *in vitro* PBMC cultures from subgroups of household contacts and leprosy patients categorized according to operational classification

**Figure 7:**
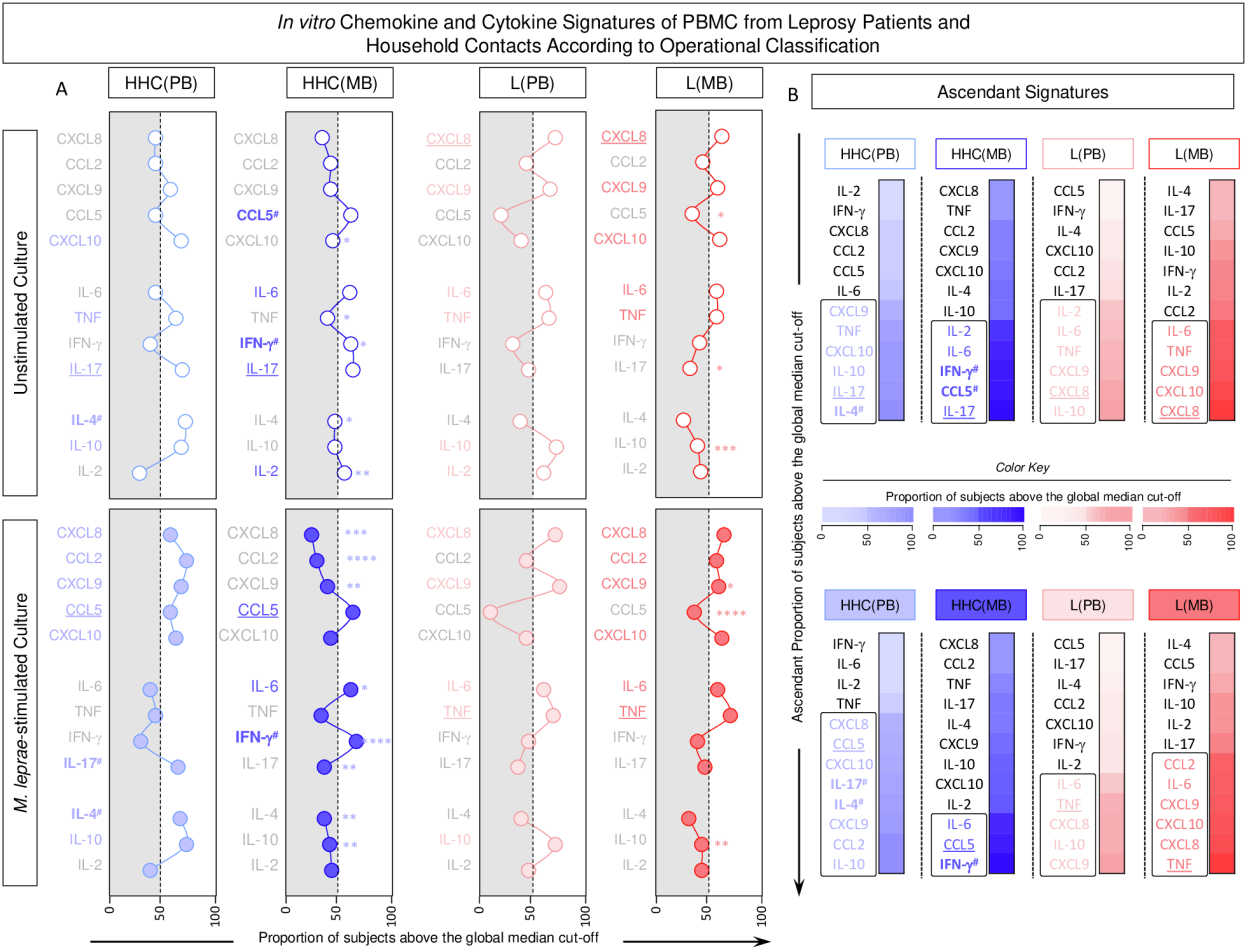
*In vitro* chemokine and cytokine signatures of PBMC from Leprosy Patients and Household Contacts according to operational classification. The levels of chemokines (CXCL8, CCL2, CXCL9, CCL5, CXCL10) and cytokines (IL-6, TNF, IFN-γ, IL-17, IL-4, IL-10 and IL-2) were measured in the supernatant from *in vitro* cultured PBMC from leprosy patients [L(PB) = 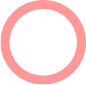; 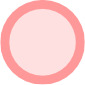, n=23 and L(MB) = 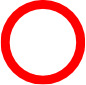; 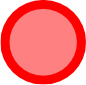, n= 56] and household contacts [HHC(PB) = 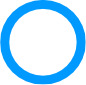; 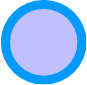, n=20 and HHC(MB) = 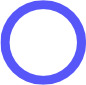; 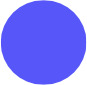, n= 68] subgroups. Data were obtained in the absence of exogenous stimuli (Unstimulated Culture = open circles) and the presence of M. leprae antigen stimuli (M. leprae-stimulated Culture = filled circles). Cytometric Beads Array (CBA) performed quantitative analysis of chemokines and cytokines according to manufacturer instructions. The results are reported as chemokine and cytokine signatures as described in the methods. Continuous variables expressed in pg/mL were converted into categorical data using the intrinsic median values of unstimulated culture or M. leprae stimulated culture as the cut-off to identify volunteers with low and high soluble mediators as described in methods. **(A)** The proportion of subjects (%) with chemokine and cytokine levels above the intrinsic cut-off was calculated for each study subgroup, and data are presented as line charts. The chemokines and cytokines with the proportion of subjects above the 50^th^ percentile were underscored and considered as increased levels were underscored by color font format. Intragroup comparisons between MB vs PB was carried out by Chi-square and significant differences indicated by * (p<0.05), ** (p<0.01), *** (p<0.001) and **** (p<0.0001). **(B)** The chemokines and cytokines signatures were assembled as ascendant signatures and subgroup-selective soluble mediators underscored by #. Colormaps illustrate the comparison amongst subgroups using a color key based on the proportion of subjects above the 50^th^ percentile (0/50/100).

The chemokine and cytokine signatures were assembled for HHC and L subgroups based on categorical data transformation using the specific cut-offs to calculate the proportion of subjects with high levels of soluble mediators as described in the methods. The results are presented in **Figure 7**. Data analysis pointed out a set of soluble mediators with the proportion of subjects above the 50^th^ percentile (**Figure 7A**, colored font). Further analysis identified intra-group common and selective soluble mediators for distinct study subgroups (**Figure 7B**). Data from unstimulated cultures revealed that IL-17 levels were elevated in HHC, independent of the operational classification, whereas CCL5 and IFN-γ were selectively increased in HHC(MB). Additionally, L subgroups showed higher levels of CXCL8 despite the operational classification. Comparative analyses of ascendant signatures from M. leprae-stimulated cultures revealed that CCL5 was a standard feature for HHC, while IL-17 and IL-4 were selectively increased in HHC(PB). Moreover, IFN-γ remained a selective HHC(MB) attribute. Data analysis also demonstrates that TNF was increased in both L subgroups (**Figure 7B**).

### Chemokine and cytokine networks in household contacts and leprosy patients according to operational classification

**Figure 8:**
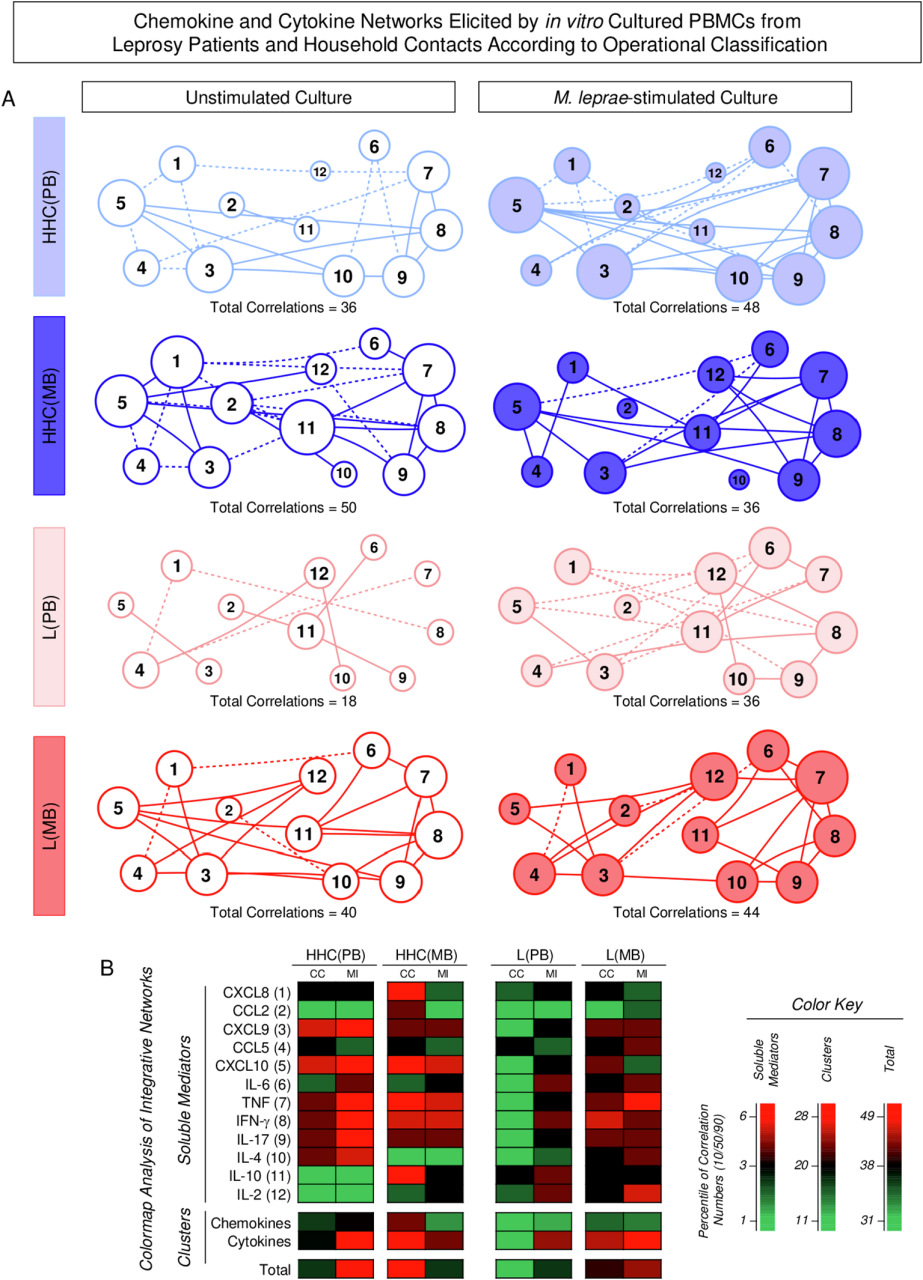
Chemokine and cytokine networks elicited by *in vitro* cultured PBMCs from Leprosy Patients and Household Contacts according to operational classification. The levels of chemokines (CXCL8, CCL2, CXCL9, CCL5, CXCL10) and cytokines (IL-6, TNF, IFN-γ, IL-17, IL-4, IL-10 and IL-2) were measured in the supernatant from *in vitro* cultured PBMC from leprosy patients [L(PB) = 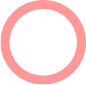; 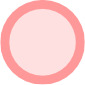, n=23 and L(MB) = 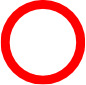; 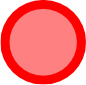, n= 56] and household contacts [HHC(PB) = 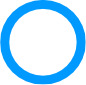; 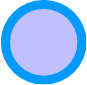, n=20 and HHC(MB) = 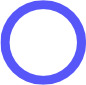; 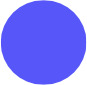, n= 68] subgroups. Data were obtained in the absence of exogenous stimuli (Unstimulated Culture = open circles) and the presence of M. leprae antigen stimuli (M. leprae-stimulated Culture = filled circles). Cytometric Beads Array (CBA) performed quantitative analysis of chemokines and cytokines according to manufacturer instructions. Integrative networks were built based on correlation analysis (Spearman rank tests) between pairs of soluble mediators, and significant correlations (p<0.05) were employed to construct networks using the open-source Cytoscape software as described in the methods. **(A)** The networks were assembled using cluster layouts with nodes representing each chemokine and cytokine (numbered as provided in the figure) and connecting lines identifying positive (“r” scores >0, continuous line) or negative (“r” scores <0, dashed line) correlations. The node sizes are proportional to the correlations between pairs of soluble mediators. Comparative analysis amongst subgroups was carried out considering the total number of correlations. **(B)** Colormaps analysis of integrative networks illustrates the comparison amongst subgroups using a color key based on the percentile distribution (10^th^/50^th^/90^th^) of correlation numbers calculated for soluble mediator, chemokine, and cytokine clusters or the total number of correlations.

Integrative networks were assembled to evaluate the interactive profile between chemokines and cytokines in the PBMC culture supernatant based on the correlation interplay between pairs of soluble mediators. The results are presented in **Figure 8**. Data analysis demonstrated that in Unstimulated cultures, the number of correlations is higher in Multibacillary subjects as compared to Paucibacillary, [HHC(MB) = 50; HHC(PB) = 36 and L(MB) = 40 and L(PB) = 18] (**Figure 8A**). In the M. leprae-stimulated cultures, the number of correlations remains higher in L(MB) as compared to L(BP) [L(MB) = 44 and L(PB) = 36] but is lower in HHC(MB) as compared to HHC(PB) [HHC(MB) = 36; HHC(PB) = 48] (**Figure 8A**). Color map analysis further corroborates these findings, as observed in the total number of correlations, the intra-cluster connectivity, and the analysis of single patterns of soluble mediators (**Figure 8B**). Detailed description of correlations between pairs of chemokines and cytokines together with the Spearman rank “r” scores are provided in the Supplementary **Figure 1**.

### Summary of changes in chemokine and cytokine profile in household contacts and leprosy patients according to operational classification

The classification of household contacts (HHC) and leprosy patients (L) based on operational classification (paucibacillary [PB] and multibacillary [MB]) reveals distinct immune signatures that highlight key differences in the cytokine and chemokine profiles. These analysis consistently underscore the critical roles of specific biomarkers in shaping the immune response to M. leprae infection, with notable variations between PB and MB subgroups.

**Figure 9:**
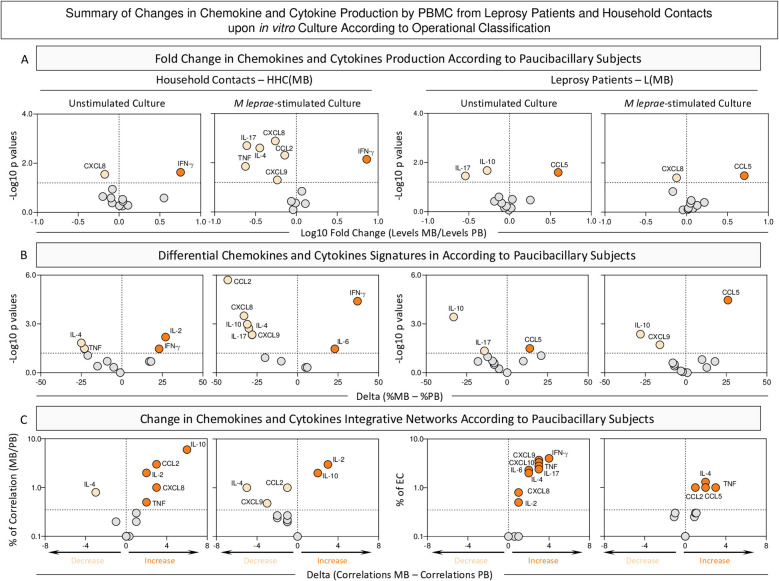
Summary of changes in chemokine and cytokine production by PBMC from Leprosy Patients and Household Contacts according to operational classification upon *in vitro* culture. The levels of chemokines (CXCL8, CCL2, CXCL9, CCL5, CXCL10) and cytokines (IL-6, TNF, IFN-γ, IL-17, IL-4, IL-10 and IL-2) were measured in the supernatant from *in vitro* cultured PBMC from leprosy patients [L(PB), n=23 and L(MB), n= 56] and household contacts [HHC(PB), n=20 and HHC(MB), n= 68] subgroups. Data were obtained in the absence of exogenous stimuli (Unstimulated Culture) and the presence of M. leprae antigen stimuli (M. leprae-stimulated Culture). Quantitative analysis of chemokines and cytokines was carried out by Cytometric Beads Array **(CBA)** according to manufacturer instructions. **(A)** Major changes observed in soluble mediators amongst subgroups according to operational classification were summarized considering the fold changes in concentrations detected in PBMC culture supernatants from MB according to PB subjects [Fold Change in concentration HHC(MB)/HHC(PB) or L(MB)/L(PB)]. Log10 of Fold Change values (magnitude) was combined with the -Log10 p values (significance) obtained by Dunn’s post-test for multiple comparisons. **(B)** Differences in chemokines and cytokines signatures were assessed according to PB subjects [Delta in % = %HHC(MB) – %HHC(PB) or %L(MB) – %L(PB)]. Log10 of Delta values (magnitude) was combined with the -Log10 p values (significance) obtained by Chi-square comparisons. **(C)** Differences in chemokines and cytokines network correlations were assessed according to PB subjects [Delta in correlation numbers = HHC(MB) – HHC(PB) or L(MB) – L(PB)]. Delta values (magnitude) were combined with the % of correlations from EC (threshold = decrease or increase > 33%). In all cases, significant decrease (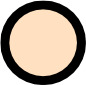), increase (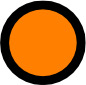), or non-significant changes (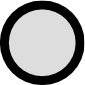) are highlighted on each chart distribution.

The **Figure 9** summarizes the main changes identified in the chemokine and cytokine profile of HHC and L upon classification into subgroups according to operational classification. The analysis of fold changes and shifts of soluble mediator signatures and integrative networks was performed in Multibacillary HHC and L subgroups according to Paucibacillary subjects.

The analysis of fold changes indicated that, regardless of the culture condition, increased levels of IFN-γ were observed in HHC(MB). Conversely, the L(MB) subgroup exhibited elevated CCL5 levels, independent of the culture condition. (**Figure 9** – top panels).

Data from soluble mediator signatures further corroborated the increase of IFN-γ in HHC(MB) regardless of the culture condition. Data from the L(MB) subgroup confirmed the increased levels of CCL5 despite the culture condition (**Figure 9**, middle panels).

The analysis of correlation networks demonstrated that in unstimulated conditions, the correlation numbers between soluble mediators in MB subgroups increase compared to PB. However, upon M. leprae stimuli, while a reduction in the number of correlations was observed in HHC(MB), an increase in correlations was found for L(MB) (**Figure 9**, bottom panels).

An integrative analysis of changes in chemokines and cytokines highlights distinct immune response profiles that differentiate HHC(MB) and L(MB) from PB subgroups (**Supplementary Figure 2**). Key biomarkers emerge, defining the common and selective immunological landscapes of each group. An increase in TNF stands out as common marker of inflammation in HHC group across all culture conditions. Conversely, a decrease of CCL5 and IL-4 appears prominently in the L group, regardless the culture condition (**Supplementary Figure 2**).

## Discussion

Leprosy persists as a relevant public health problem in several countries in Asia, Africa, and Latin America (1). The Host’s immune response plays a critical role in the pathophysiology of leprosy, and genetic factors influence the clinical course of the disease (18). Using immunological biomarkers as diagnostic/prognostic tools is a relevant complementary strategy to classify leprosy patients into distinct clinical forms and support early clinical interventions, prompt initiation of treatment, and effective leprosy control (19). Queiroz and colleagues have compared several immunological features of leprosy index cases and household contacts to identify immune response biomarkers and provide evidence of subclinical infection in leprosy household contacts (7). Although several immunological parameters displayed similar profile index cases and household contacts, which suggests the lack of immunological distance between them, some biomarkers could be used as subclinical infection predictors in contacts (7). On the other hand, it has been proposed that no specific test will predict who will develop leprosy after exposure (20). Indeed, the observation that high exposure to M. leprae can lead to a decrease in host resistance was first described in 1973 (21). Attempts are still necessary to increase the limited clusters of immune parameters already available for predictive diagnosis and laboratory monitoring. In this study, we have used a complementary approach to thoroughly map out the landscape of chemokine and cytokine profiles. Our main goal was to establish correlations between these biomarker patterns and specific clinical classifications among leprosy patients and their household contacts using an *in vitro* model of antigen-specific stimuli for peripheral blood mononuclear cells. Soluble mediator analysis, presented as biomarker concentrations in pg/mL, evaluated as continuous variables, revealed that leprosy patients and HHC exhibited elevated levels of TNF upon M. leprae-stimulated culture compared to EC. However, the distinguishing biomarkers for the L group were CXCL9, CXCL10, and TNF, while IFN-γ characterized HHC. Furthermore, the Venn diagram analysis of M. leprae-stimulated cultures illustrated IL-17, IL-4, and IL-2 as distinctive attributes for EC. These findings highlight an upregulation of IFN-γ in household contacts and CXCL10 in leprosy patients. The cytokine production in active lymphocytes and monocytes, along with the differentiated production of chemokines, appears to play a significant role in shaping the immune response and influencing various clinical aspects of the disease. Research indicates that the production of TNF in monocytes and IFN-γ in lymphocytes supports cellular interaction and regulates the activation of chemokines (22). Ferreira and colleagues have demonstrated that a decrease in CXCL10 levels in sera correlated with an absence of a reduction in bacillary load upon release, implying that CXCL10 could serve as a promising marker for monitoring treatment efficacy in multibacillary patients (23). As household contacts of L(PB) and L(MB) are exposed to distinct M. leprae antigenic stimuli levels, their adaptive immune response likely differs in components and magnitude. Moreover, it has been suggested that IFN-γ production induced by M. leprae can identify individuals highly exposed to the bacterium, thus potentially at a higher risk of developing clinical disease and/or transmitting the pathogen (17). We observed significantly higher production of IFN-γ in HHC(MB), consistent with previous findings that individuals highly exposed to M. leprae show elevated IFN-γ responses (17, 18). This highlights the potential role of IFN-γ in identifying individuals at risk of developing leprosy. The elevated IFN-γ production in response to M. leprae antigen stimuli *in vitro* among contacts of leprosy patients suggests a heightened frequency of sensitization. Analysis of fold changes in soluble mediator concentrations revealed decreased levels of CXCL8 and CCL2 and increased levels of TNF and IL-10 for HHC, regardless of the culture condition. In contrast, the L group exhibited a consistently elevated TNF profile irrespective of the culture condition. Furthermore, data from soluble mediator signatures support the idea that the L group demonstrated sustained TNF persistence. Bezerra-Santos and colleagues have shown that TNF levels correlate with the clinical outcome of patients with leprosy (24). Elevated circulating levels of TNF are observed in the multibacillary form of leprosy, suggesting a significant involvement of this biomarker in the pathogenesis of the disease. These findings highlight the potential of TNF as a promising biomarker to aid in diagnosing leprosy complications (24). A comparative analysis of subgroups comprising HHC and Leprosy patients revealed distinct patterns. Specifically, among HHC(MB), higher levels of IFN-γ and lower levels of CXCL8 were observed compared to HHC(PB). Conversely, L(PB) exhibited elevated levels of IL-17 and IL-10, alongside decreased levels of CCL5, compared to L(MB). Employing the ascending signature analysis curve of the groups of patients with leprosy and household contacts could reveal an significant changes in the expression of most of the biomarkers evaluated, especially TNF and IFN-g levels, that were observed when comparing L(MB) and HHC(MB) to the EC group, suggesting a graded immune response based on exposure levels. These data underscored the concept that leprosy should not be viewed solely as a skin condition but as a significant and systemic immunological condition (25). Several studies have previously documented the augmentation of pro-inflammatory cytokines in HHC(MB) (26, 27). The precise relationship between pro-inflammatory cytokines and exposure to M. leprae remains controversial. Martins and colleagues have proposed an inverse correlation between IFN-γ levels and the degree of M. leprae exposure (28). Additionally, Sampaio and colleagues have reported higher M. leprae-induced IFN-γ production in multibacillary contacts compared to paucibacillary contacts (29). Moreover, *in vitro* IFN-γ production in response to M. leprae antigen stimuli is elevated among contacts of leprosy patients, suggesting a high frequency of sensitization. de Carvalho and colleagues have demonstrated continuous exposure of multibacillary contacts to live M. leprae bacilli that downregulates the specific cellular immune response against the pathogen (22). It is plausible that the extent of response to M. leprae antigen primarily depends on the duration of contact and the infectiousness of leprosy patients. Our findings support the hypothesis that greater exposure increases the predisposition to develop the disease. Therefore, the prominent pro-inflammatory profile observed in HHC(MB) may indicate early subclinical infection. Network analyses of soluble mediator interactions were used to offer a panoramic view of the results and their interactions in leprosy patients, household contacts, and endemic control subjects. When the biomarkers were analyzed for network interactions, it became evident that the disease significantly altered their connections, exhibiting distinct patterns in each group. Data analysis revealed that correlations were higher in Multibacillary subjects than in Paucibacillary subjects but lower in HHC(MB) compared to HHC(PB). We posit that the variability observed in the soluble mediator networks among different groups aligns with the intricate clinical spectrum through which leprosy can manifest, ranging from minor skin lesions to severe cases of the disease. The analysis of fold changes revealed that regardless of the culture condition, decreased levels of CXCL8 and increased levels of IFN-γ were observed for HHC(MB). Conversely, the L(MB) subgroup exhibited higher levels of CCL5 irrespective of the culture condition. Soluble mediator signatures further supported these findings, demonstrating that IFN-γ was increased and IL-10 was decreased in HHC(MB) regardless of the culture condition. Moreover, the analysis of integrative networks indicated that under unstimulated conditions, there was an overall increase in correlations between soluble mediators in MB subgroups compared to PB subgroups. However, upon stimulation with M. leprae, while a reduction in the number of correlations was observed in HHC(MB), an increase in correlations was found for L(MB). It is noteworthy to emphasize the predominantly pro-inflammatory profile of immunological biomarkers exhibited by individuals in the HHC group. Some authors advocate for including HHC in health programs, even proposing chemoprophylaxis to prevent future clinical disease, impairment, and disability (22).

Nonetheless, it is important to mention that the present study has some limitations, particularly regarding the number of samples evaluated. Moreover, considering the influence of age and sex in the levels of chemokines and cytokines (30, 31), data analysis adjustments for these variables should be considered in future investigations. Further research with larger cohorts will be essential to confirm and extend these findings. Despite being based on a single experiment, this study offers new insights into the biomarkers associated with the immune response to M. leprae.

Together, the overall findings contributed with additional insights into the immunological features associated with clinical aspects of leprosy patients and their household contacts. Notably, the present study confirms and re-enforces that TNF, CXCL8, and IFN-γ are promising immunological markers in the avenues for diagnostic investigation. In general, the *in vitro* M. leprae-stimulation of PBMCs provided additional differences in the TNF levels for comparison between L vs HHC as well as between HHC(PB) vs HHC(MB). Additionally, differences in the CXCL8 levels between L(PB) vs L(MB) was only observed in M. leprae-stimuli condition. These findings demonstrated the relevance of performing M. leprae-stimulation to potentialize the differences between groups. Additional experiments will be necessary to further validate and expand upon these observations. Collectively, our data underscore the value of exploring cell-mediated immune components as potential biomarkers to improve the early detection of subclinical leprosy in household contacts.

## Conclusions

Our data strongly suggests that immune response biomarkers can serve as valuable complementary tools for diagnostic and prognostic purposes while indicating that household contacts may harbor subclinical infection, particularly those categorized as HHC(MB), exhibiting significant antigen-specific cytokine secretion levels by peripheral blood cells. It is crucial to monitor these individuals for disease development in the future. Early detection of leprosy cases and prompt initiation of chemotherapy are essential strategies for reducing the incidence of new cases and preventing transmission. As the household contacts represent a group of subjects at high risk of developing disease due to their proximity to a potential source of infection, we propose that antigen-specific cytokine secretion detection can be helpful as a preventive strategy. This approach can facilitate the follow-up of leprosy household contacts, allowing for the confirmation or exclusion of subclinical infection. The measurement of soluble biomarkers, such as cytokines and chemokines, could be included in the routine laboratory monitoring portfolio for household contacts in endemic regions. Regular assessment of TNF and IFN-γ levels could help detect immune responses indicative of subclinical infection, enabling early intervention before the onset of clinical symptoms.Regular measurement of TNF and IFN-γ levels could help detect immune responses indicative of subclinical infection, allowing for early intervention before the onset of clinical symptoms

## Data Availability

The original contributions presented in the study are included in the article/**Supplementary Material**. Further inquiries can be directed to the corresponding authors.

## Supplementary material

Supplementary Figure 1 Correlograms of chemokine and cytokine networks elicited by *in vitro* cultured PBMCs from Leprosy Patients and Household Contacts according to operational classification. The levels of chemokines (CXCL8, CCL2, CXCL9, CCL5, CXCL10) and cytokines (IL-6, TNF, IFN-γ, IL-17, IL-4, IL-10, and IL-2) were measured in the supernatant from *in vitro* cultured PBMC from leprosy patients [L = , n=79; L(PB) = , n=23; L(MB) = , n=56], household contacts [HHC = , n=91; HHC(PB) = , n=20; HHC(MB) = , n=68] and endemic controls [EC = , n=87]. Data were obtained in the absence of exogenous stimuli (Unstimulated Culture) and the presence of M. leprae antigen stimuli (M. leprae-stimulated Culture). Cytometric Beads Array (CBA) performed quantitative analysis of chemokines and cytokines according to manufacturer instructions. Correlograms were built based on the “r” scores of significant correlations (p<0.05) from Spearman rank test between pairs of soluble mediators. Correlation matrices were assembled using circle layouts with nodes representing each pair of chemokine and cytokine (numbered as provided in the figure) identifying positive (“r” scores >0, red circles) or negative (“r” scores <0, blue circles) correlations. The circle sizes are proportional to the “r” score modular values.

Supplementary Figure 2 Integrative overview of common and selective changes in chemokine and cytokine produced *in vitro* cultured PBMCs from Leprosy Patients and Household Contacts according to operational classification. The levels of chemokines (CXCL8, CCL2, CXCL9, CCL5, CXCL10) and cytokines (IL-6, TNF, IFN-γ, IL-17, IL-4, IL-10 and IL-2) were measured in the supernatant from *in vitro* cultured PBMC from leprosy patients [L, n=79; L(PB), n=23 and L(MB), n= 56] and household contacts [HHC, n=91; HHC(PB), n=20 and HHC(MB), n= 68] subgroups. Data were obtained in the absence of exogenous stimuli (Unstimulated Culture) and the presence of M. leprae antigen stimuli (M. leprae-stimulated Culture). Quantitative analysis of chemokines and cytokines was carried out by Cytometric Beads Array (CBA) according to manufacturer instructions. Major changes in soluble mediators observed between HHC vs EC and L vs EC, as well as amongst subgroups according to operational classification [HHC(MB) vs HHC(PB) or L(MB) vs L(PB)] were summarized considering three distinct approaches: i) fold changes in concentrations [pg/mL), ii) differences in signatures [% > MED] and iii) changes network correlations [# Edges]. Venn diagram analysis was applied to identify attributes with common and selective decrease (green background) or increase (red background) amongst groups. Common attributes in all or at least two approaches are underscored by white letters.
