# Supplementary figures and images for: The landscape of chemokine and cytokine is associated with the distinct clinical status of leprosy patients and their respective household contacts
Source: Front Immunol. 2024 Dec 18;15:1476450. doi: 10.3389/fimmu.2024.1476450 (PMC11688302; doi:10.3389/fimmu.2024.1476450)

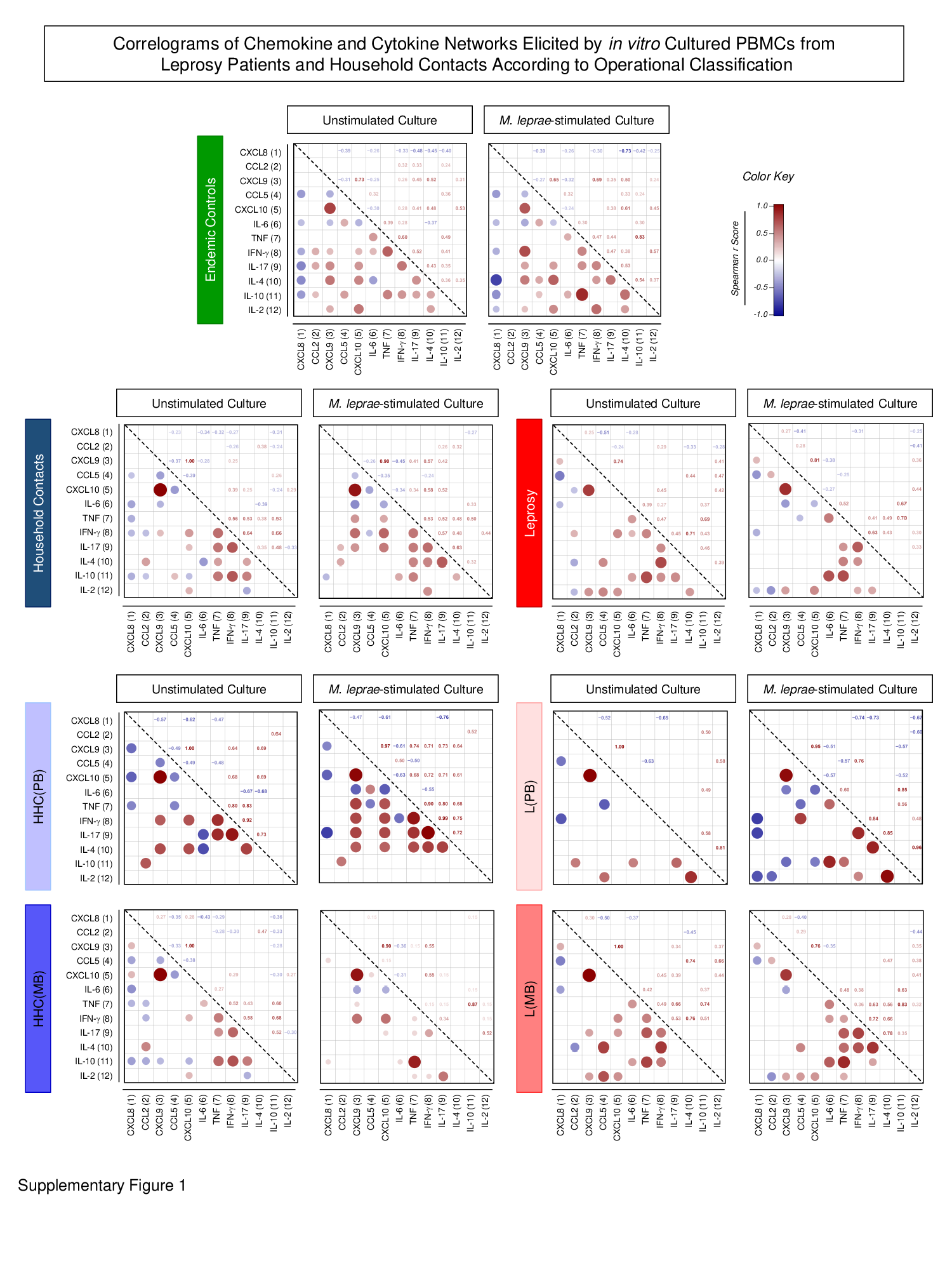

Supplement: Supplementary Figure 1 — Correlograms of chemokine and cytokine networks elicited by in vitro cultured PBMCs from Leprosy Patients and Household Contacts according to operational classification. The levels of chemokines (CXCL8, CCL2, CXCL9, CCL5, CXCL10) and cytokines (IL-6, TNF, IFN-γ, IL-17, IL-4, IL-10, and IL-2) were measured in the supernatant from in vitro cultured PBMC from leprosy patients [L = , n=79; L(PB) = , n=23; L(MB) = , n=56], household contacts [HHC = , n=91; HHC(PB) = , n=20; HHC(MB) = , n=68] and endemic controls [EC = , n=87]. Data were obtained in the absence of exogenous stimuli (Unstimulated Culture) and the presence of M. leprae antigen stimuli (M. leprae-stimulated Culture). Cytometric Beads Array (CBA) performed quantitative analysis of chemokines and cytokines according to manufacturer instructions. Correlograms were built based on the “r” scores of significant correlations (p<0.05) from Spearman rank test between pairs of soluble mediators. Correlation matrices were assembled using circle layouts with nodes representing each pair of chemokine and cytokine (numbered as provided in the figure) identifying positive (“r” scores >0, red circles) or negative (“r” scores <0, blue circles) correlations. The circle sizes are proportional to the “r” score modular values. [file Image1.tiff]

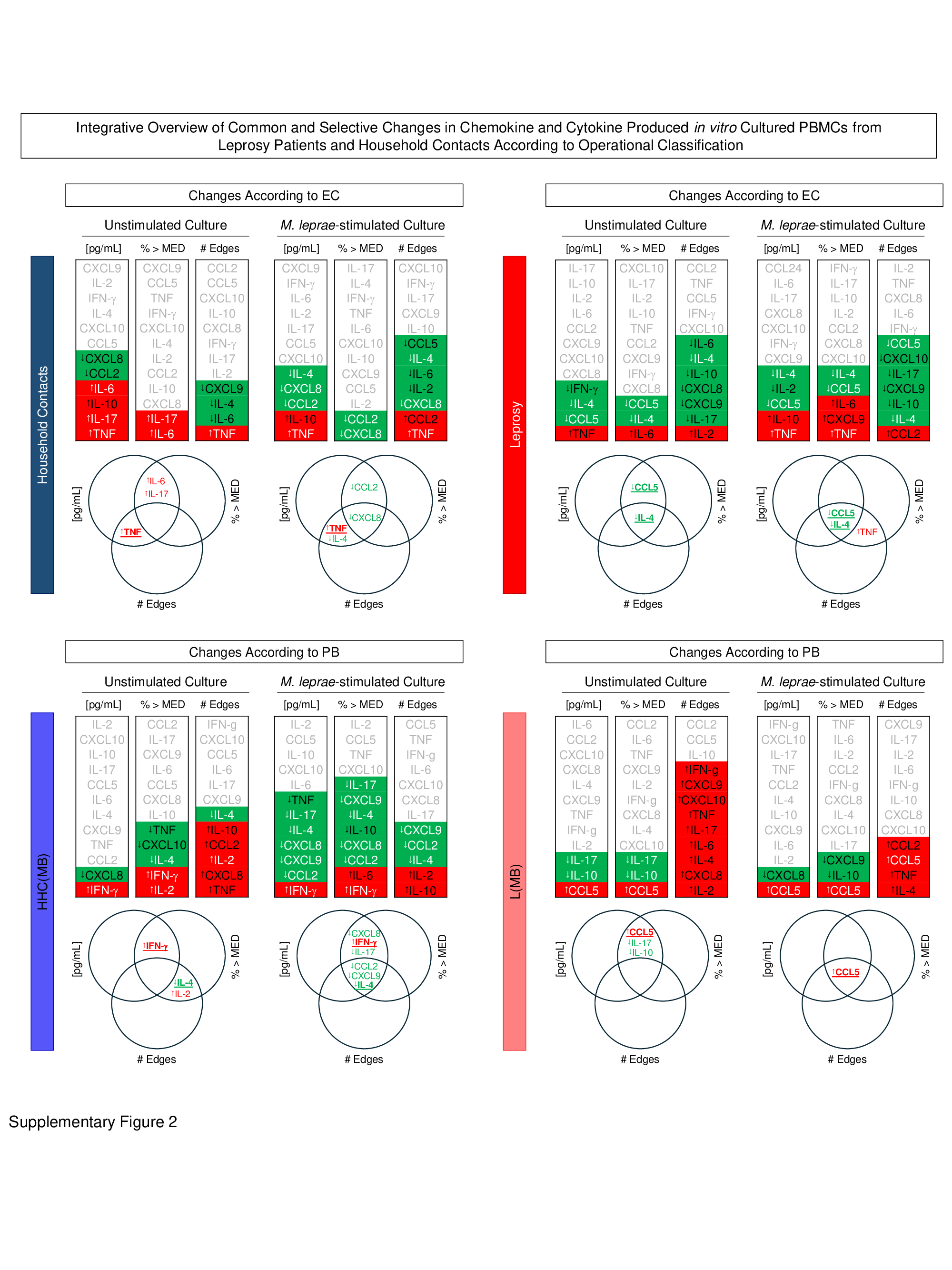

Supplement: Supplementary Figure 2 — Integrative overview of common and selective changes in chemokine and cytokine produced in vitro cultured PBMCs from Leprosy Patients and Household Contacts according to operational classification. The levels of chemokines (CXCL8, CCL2, CXCL9, CCL5, CXCL10) and cytokines (IL-6, TNF, IFN-γ, IL-17, IL-4, IL-10 and IL-2) were measured in the supernatant from in vitro cultured PBMC from leprosy patients [L, n=79; L(PB), n=23 and L(MB), n= 56] and household contacts [HHC, n=91; HHC(PB), n=20 and HHC(MB), n= 68] subgroups. Data were obtained in the absence of exogenous stimuli (Unstimulated Culture) and the presence of M. leprae antigen stimuli (M. leprae-stimulated Culture). Quantitative analysis of chemokines and cytokines was carried out by Cytometric Beads Array (CBA) according to manufacturer instructions. Major changes in soluble mediators observed between HHC vs EC and L vs EC, as well as amongst subgroups according to operational classification [HHC(MB) vs HHC(PB) or L(MB) vs L(PB)] were summarized considering three distinct approaches: i) fold changes in concentrations [pg/mL), ii) differences in signatures [% > MED] and iii) changes network correlations [# Edges]. Venn diagram analysis was applied to identify attributes with common and selective decrease (green background) or increase (red background) amongst groups. Common attributes in all or at least two approaches are underscored by white letters. [file Image2.tiff]
